# Supplementary material for: Prophylactic low-dose, bi-weekly benznidazole treatment fails to prevent Trypanosoma cruzi infection in dogs under intense transmission pressure
Source: PLoS Negl Trop Dis. 2022 Oct 31;16(10):e0010688. doi: 10.1371/journal.pntd.0010688 (PMC9648846; doi:10.1371/journal.pntd.0010688)
Supplement: S1 Fig — (PDF) [file pntd.0010688.s001.pdf]

Figure S1

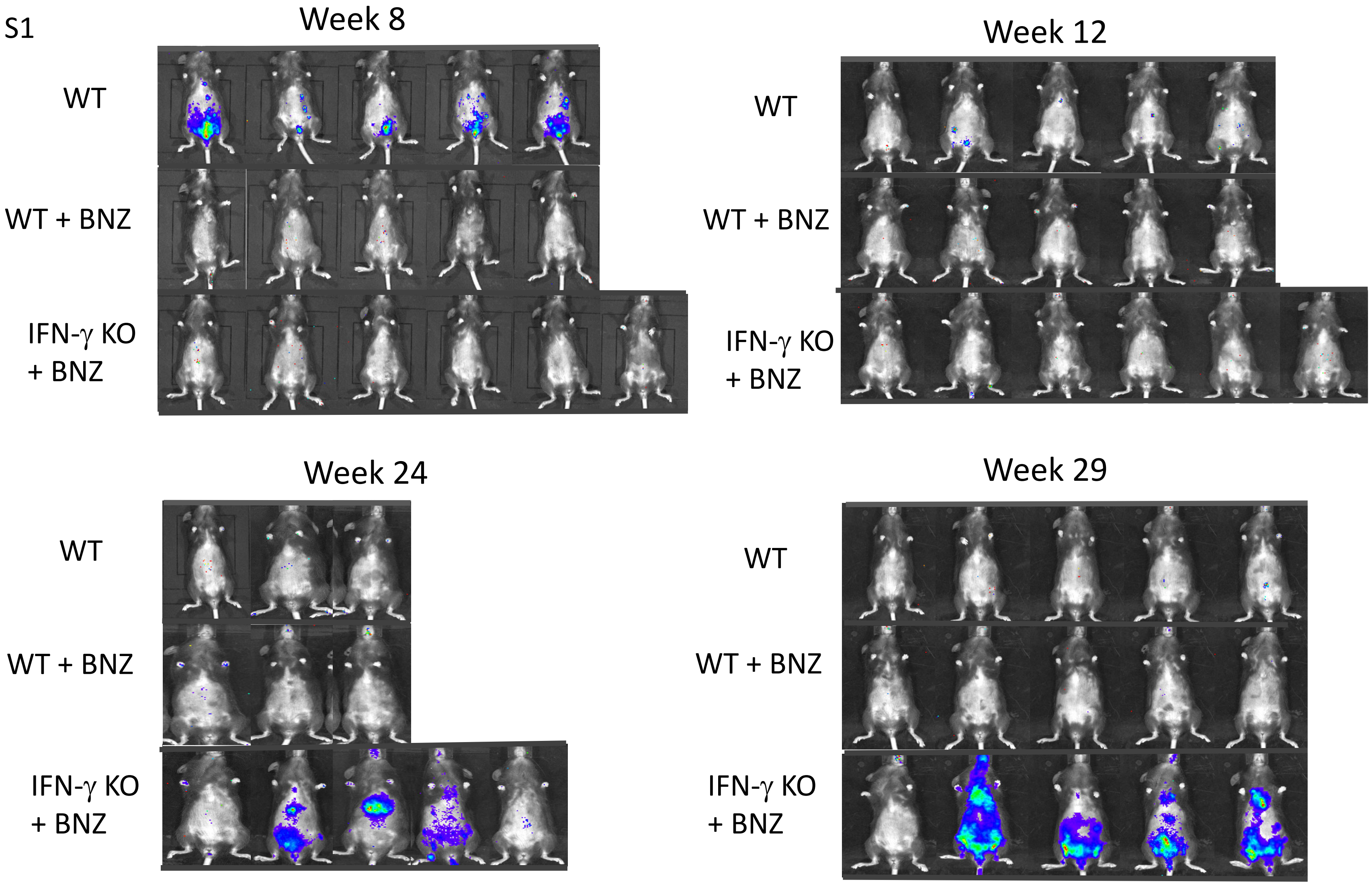
Week 8
WT
WT + BNZ
IFN-γ KO
+ BNZ
Week 12
WT
WT + BNZ
IFN-γ KO
+ BNZ
Week 24
WT
WT + BNZ
IFN-γ KO
+ BNZ
Week 29
WT
WT + BNZ
IFN-γ KO
+ BNZ
